# Muscle-Cooling Intervention to Reduce Fatigue and Fatigue-Induced Tremor in Novice and Experienced Surgeons: A Preliminary Investigation

**DOI:** 10.1055/s-0036-1594246

**Published:** 2016-11-14

**Authors:** Lauren Jensen, Michael Dancisak, James Korndorffer

**Affiliations:** 1 Doctoral candidate in Aging Studies, Tulane University, New Orleans, Louisiana; 2 Department of Biomedical Engineering, Tulane University, New Orleans, Louisiana; 3 Center for Advanced Medical Simulation and Team Training, Tulane University, New Orleans, Louisiana

**Keywords:** muscle fatigue, surgeon fatigue, muscle cooling, tremor reduction, fatigue, physiologic tremor, surgical tremor

## Abstract

A localized, intermittent muscle-cooling protocol was implemented to determine cooling garment efficacy in reducing upper extremity muscular fatigue and tremor in novice ( *n* = 10) and experienced surgeons ( *n* = 9). Subjects wore a muscle-cooling garment while performing multiple trials of a forearm exercise and paired suturing task to induce muscular fatigue and exercise-induced tremor. A reduction in tremor amplitude and an extension in time to fatigue were expected with muscle cooling as compared with control trials. Each subject completed an intervention session (5°C cooling condition) and a control session (32°C or thermal neutral condition). A paired samples *t* test indicated that tremor amplitude was significantly reduced ( *t* [8] = 1.89458; *p* < 0.05) in experienced surgeons in two dimensions (up and down, and back and forth). Tremor amplitude was reduced in novice surgeons but the effect was not significant. Time to fatigue and suture time improved in both cohorts with muscle cooling, but the effect did not reach significance. Results from the pilot work suggest muscle cooling as an intervention for reduction of fatigue and tremor is very promising, warranting further investigation. Surgical specialties that require prolonged procedures might benefit more from this intervention.

Muscular fatigue and fatigue-induced physiologic tremor can encumber both novice surgeons as well as experienced surgeons during long or demanding surgeries. Muscle cooling has been shown to reduce muscular fatigue levels and physiologic tremor amplitude. 1 2 Cooling vests and garments have been available to operating room (OR) staff for some time now, but the investigation of their efficacy has been limited. As the current surgical workforce ages, 3 a larger portion of the medical community is calling for a more objective evaluation of surgeons nearing retirement. 4

Increases in limb tremor amplitude were reported after fatigue-induced exercise and after localized muscle heating. 2 5 In contrast, limb cooling and intermittent ischemia has been shown to reduce limb tremor amplitude. With age, skeletal muscle mass, muscle strength, and quality of muscle all decline ∼5% per decade in both static and dynamic muscle strength measures. 6 Reduction in muscle mass is a prominent contributor to chronic muscle fatigue. A recent study found that a group of active, well-trained older men (average age of 70 years) who exercised regularly as compared with sedentary cohorts have better preserved muscle structure and function. 7 The encouraging prospect that age-related declines in muscle function and structure may be attenuated by regular weekly exercise comes with the limitation that there will be periods of postactivity fatigue that may also increase tremor.

Muscle cooling is a nonpharmacologic and nonsurgical modality to reduce physiologic tremor and muscular fatigue for endurance exercises. 2 8 Therapeutic muscle cooling in athletes and other high-performance professionals can be achieved through a variety of noninvasive methods during or after periods of exertion. Advances in garment design and material science have allowed for the use of improved cooling methods that expedite muscle recovery and reduce inflammation during exercise (or at discrete rest intervals) to extend time to regional muscle fatigue or attenuate physiologic tremor. Muscles have an optimal temperature range outside which muscle function can be impaired. The cooling garment was designed to cool target muscle areas to a minimum of 18°C (lower limit for thermoreceptors and nociception threshold). 9 Muscles and cutaneous tissue sense temperature changes through thermoreceptors that transmit signals to the hypothalamus where the information is integrated with both conscious and unconscious response centers in the brain.

Increased levels of fatigue and tremor in aging surgeons are commonly discussed in the medical community; however, experimental evidence delineating these physiologic issues facing older surgeons is almost nonexistent. For example, the Aging Surgeon program, initiated in February 2014 as a means for surgeons to demonstrate and document baseline skills and periodically re-evaluate their surgical skill set, has seen very limited participation. 5 Although several survey studies have been conducted regarding the impact of aging upon surgeon performance, there is a marked lack of physical and biometric data on this topic. This research pilots methodology that investigates the ability of a cooling garment to reduce fatigue and fatigue-induced tremor in both novice and experienced surgeons using simulated OR tasks. No significant differences were anticipated between novice and experienced currently practicing surgeons for physiologic tremor or fatigue measures.

## Background

Surgical fatigue syndrome (SFS) describes the normal decline observed in surgical performance after 4 hours of surgery. 10 Characteristics of SFS include mental exhaustion, increased irritability, decreased surgical judgment, and decreased dexterity. Preventative measures for SFS include rest periods (at least 10 minutes for every 2 hours) and a shared workload. Overuse syndromes (tenosynovitis, repetitive strain injury, cumulative trauma disorder, acute carpal tunnel syndrome) frequently occur from repetitive minimally invasive surgeries. Increased effort prior to fatigue has been documented by electromyography of forearm muscles, indicating increased risk for injury that may occur prior to conscious awareness of muscle fatigue. 11 Anatomically, risk for injury develops from nerve compression at both the superficial terminal branch and the dorsal digital branches of the radial nerve and the palmar digital nerves when manipulating laparoscopic instruments. Long-term manifestations of these actions may result in neuropraxia, axonotmesis, or neurotmesis. Although superficial cooling would slow nerve-firing rates, the muscle-cooling protocol implemented in this research cools at a more proximal region than the nerve branches mentioned.

Although several different methods for defining or measuring fatigue are available, this research uses a “time to functional fatigue” measure, which is a realistic and functional measure of the ability of a subject to perform a continuous task over a defined period of time with the corresponding change in range of motion. Other measures of fatigue include shifts in electromyography frequency over the duration of a task, biomarkers that assay lactate levels pre- and postperformance, and changes in kinematics and kinetics. 12

Variation in the onset and recovery from fatigue are known to be different between dominant versus nondominant task-specific muscle groups. Previous research has shown the right trapezius fatigues more than the left trapezius. Additionally, fatigue differences have been noted between the left and right mid deltoid and erector spinae muscles for surgeons in urology. 13 Surgeons from different specialties experience various levels of fatigue of the brachioradialis and mid-deltoid muscle. (Slack demonstrated that the brachioradialis muscle fatigues 1.5 times as fast as the mid-deltoid muscle. 14 ) Knowledge of differential fatigue between muscle groups is essential to guide cooling garment designs and modifications.

Physiologic tremor is an ever-present obstacle to many surgical residents and an ongoing challenge for practicing surgeons, especially those specialties that perform microsurgery. 15 In a double-masked, placebo-controlled study, 16 ophthalmic surgeons ( *n* = 17) deviated from their baseline tremor by +15% after taking a placebo and +31% after ingesting caffeine. In contrast, after taking propranolol there was a 22% decline from baseline tremor. Other work supports the significant effect of medicating with β-blockers (propranolol 1 hour prior to surgery) and specifically how it reduces tremor and anxiety levels in ophthalmic surgeons as compared with controls taking placebos. 17 Although medication studies have shown promising results, exploring nonpharmacologic intervention options could provide reduced fatigue and/or tremor in aging surgeons. One nonpharmacologic modality that has been studied is the proximity of exercise prior to surgery. Significant increases in tremor were observed after upper body strength or resistance exercises and aerobic exercise in surgeons. 18 Limb tremor amplitude increases with exercise-induced fatigue and heating, 2 5 whereas limb cooling has been shown to reduce tremor amplitude. Current options to reduce physiologic tremor include the reduction or cessation of caffeine intake prior to surgery, medication such as propranolol, nonbiological approaches such as meditation, the use of tremor-canceling devices, and reducing limb temperature. This pilot work focuses on the later as it examined changes in physiologic tremor and a defined time to fatigue metric utilizing a cooling garment.

## Methods

The novice surgeon cohort ( *n* = 10) included resident surgeons up until 5 years after completing their residency (maximum age of 35 years; average age 31.2 ± 2.1 years; age range 28 to 35), and the experienced cohort ( *n* = 9) included experienced surgeons aged 50 and above (required ≥ 10 year experience; average 60 ± 10 years; age range 52 to 79). Cohorts were only separated by age group and not by sex, as each subject was compared with his or her own baseline values. Surgeons were recruited through the Tulane University Medical School and throughout the Tulane surgical community. Testing took place at the Tulane Center for Advanced Medical Simulation and Team Training on the Medical School Campus. Participants were offered $50.00 as an incentive to participate, which was paid only at the completion of the study trials.

**Fig. 1:**
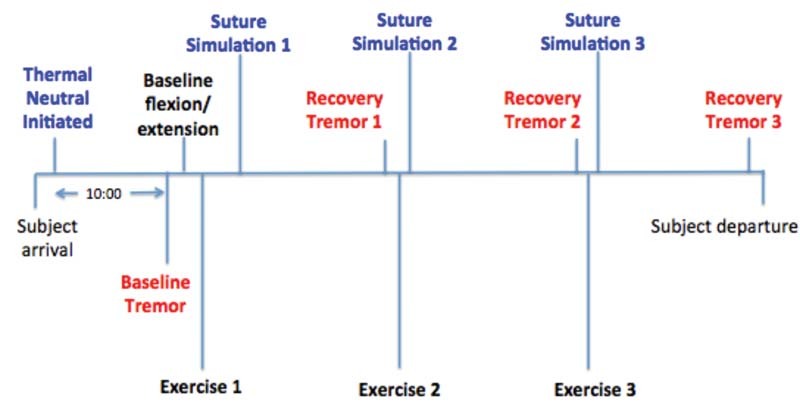
Timeline of protocol. Each subject completed two sessions (intervention and control) separated by at least 72 hours; presentation of session condition was randomized per subject. Color code: blue, suture task; red, tremor recording; black, flexion-extension exercise.

Upon arrival, each session began with an acclimatization period while wearing the cooling garment (set to thermal neutral, or 32°C). Two standard commercial chillers (NESLAB RTE-110, Chiller City Corporation, Mesa, Arizona, United States) regulated the temperature in the cooling garment. Tremor data was acquired at 200 Hz and measured with a velocity transducer (subject held hemostats in each hand with a semirigid plastic 1-mm ^2^ stylus on a ∼1-mm ^2^ moveable target). Subjects were allowed to anchor one finger of each hand on the test stand. Only time on target was counted; slips off of the needle were not included. All data were acquired and processed with the acquisition hardware (Biopac MP150, Biopac Systems, Inc., Goleta, California, United States) and corresponding Acknowledge software (version 4.2; Biopac Systems, Inc.). Experimental tremor measures reported include the percentage change from baseline of tremor amplitude for the y (up and down) and z (back and forth) dimensions. Refer to Fig. 1 for a full protocol timeline.

**Fig. 2:**
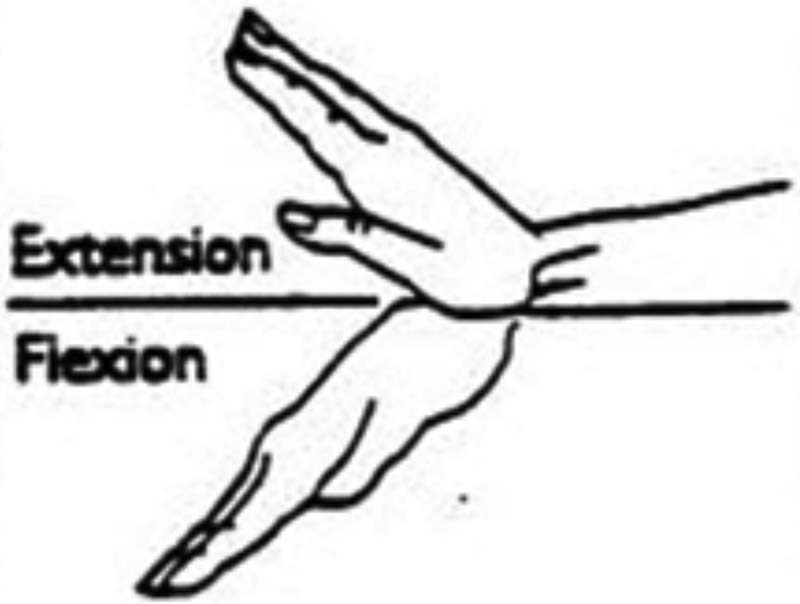
Subjects required to flex and extend their wrist while holding a 5-lb hand weight (full range of motion; view from above).

The fatiguing exercise consisted of a wrist flexion-extension activity where the subject used the dominant arm, which was flexed at the elbow 90 degrees and the wrist was pronated 90 degrees with the hand holding a 5-lb weight ( Fig. 2 ). Maximum range of motion was recorded prior to the initiation of the exercise to set baseline limits. Study participants were required to continue the flexion-extension exercise until they could no longer maintain within 10 degrees of their full range of motion for either flexion or extension for three consecutive repetitions (functional fatigue). Once subjects reached functional fatigue, they completed a suturing task comprised of five continuous running sutures, each of which was closed with a square knot with four throws. This task was timed, and subjects were informed that they have as long as they need to complete the task but they are being timed for efficiency. After completion of the task, recovery tremor was recorded (no rest period). Three trials of fatigue, rest, and recovery tremor were completed per condition. Tremor data were analyzed for percentage change in target displacement for each set of tasks. Statistical analyses were completed using a Student *t* test for paired samples (SPSS Version 22, IBM Corp., Armonk, New York, United States).

## Results

The novice surgeon cohort (age range: 28 to 35 years) reported regular participation in the following types of exercise: cardio (8), yoga (2), lifting (1), tennis (1), and kickball (1). The experienced surgeon cohort (age range: 50 to 79 years) reported regular participation in the following types of exercise: walking (5), swimming (2), lifting (2), tennis (3), and yoga (1). All subjects were right-handed (one exception was left hand dominant but sutured with the right hand; Table 1 ).

**Table 1:** Descriptive information on novice and experienced surgeon subjects

Cohort	*n*	Age (y)	Years of surgical experience	Work (h/wk)	Surgery (h/wk)	Exercise (h/wk)
Novice	10 (6 women, 4 men)	31.2 ± 2.1	3.9 ± 2.3	73.5 ± 7.4	15.0 ± 11.3	4.8 ± 3.6
Experienced	9 (all men)	60.9 ± 8.0	34.6 ± 8.7	53.9 ± 14.2	15.8 ± 6.9	7.4 ± 5.1

Note: All results are reported as average ± standard deviation.

### Physiologic Tremor

**Fig. 3:**
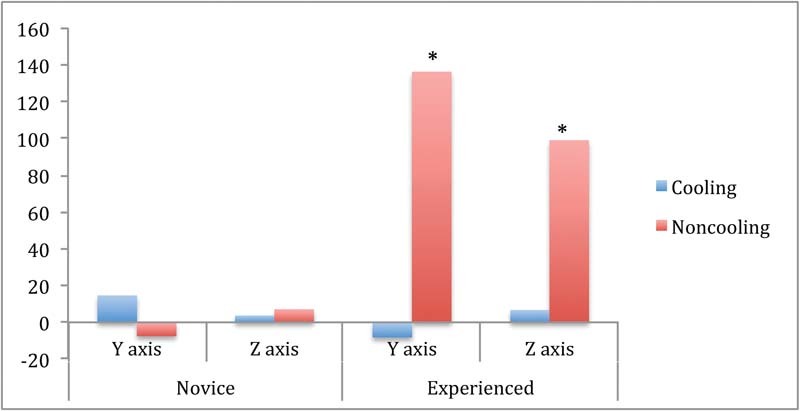
Dominant (right) hand tremor in novice and experienced cohorts (y-axis; averaged over trials). *Indicates *p* < 0.05.

Tremor in the novice surgeon cohort was highly variable, with values much below baseline, suggesting a substantial amount of adaptation to perfecting the task with repeated performance ( Fig. 3 ). Tremor values are reported as a percentage of baseline tremor (per axis). Therefore, a tremor value of 100% would be the same as the baseline tremor. Analysis indicated that experienced surgeons ( Fig. 3 ) showed a significant decrease in dominant hand tremor with the cooling condition ( *t* [8] = 1.89458; *p* < 0.05), and had a larger tremor (compared with baseline) than novice surgeons in the noncooling condition; however, their noncooling tremor remained within baseline values.

### Functional Fatigue and Suture Time

**Fig. 4:**
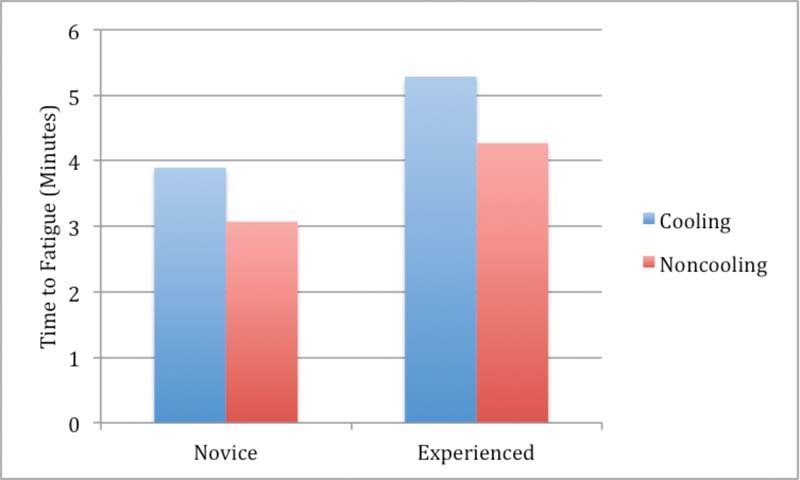
Time to functional fatigue in a weighted flexion-extension exercise, both cohorts.

**Fig. 5:**
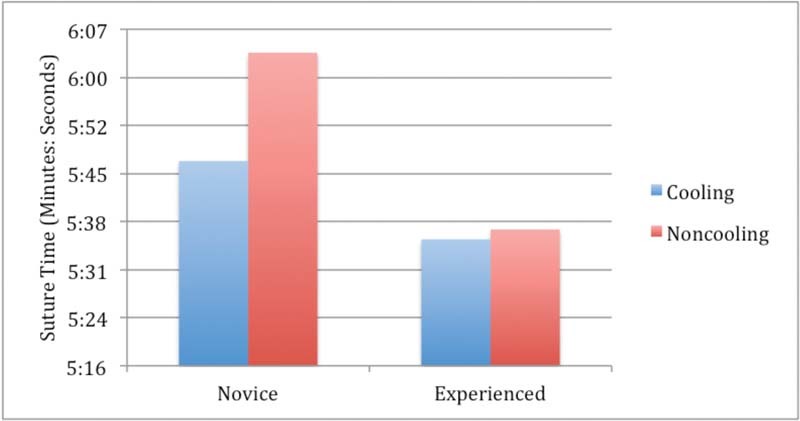
Suture time (averaged over trials), both cohorts.

For the functional fatigue (exercise) tasks, the hypothesis that cooling would result in an extended time to fatigue was supported in both cohorts ( Fig. 4 ). Suture task times ( Fig. 5 ) also showed an improvement with muscle cooling in both cohorts, though the effect did not reach significance ( Fig. 5 ).

## Discussion

Although other surgical editorials have repeatedly called for research on older surgeons, biometric evaluations of aging surgeons will remain limited until they make themselves more available as research subjects. Fatigue and tremor are inevitable and at the same time undesirable in the OR. Exercise-induced tremor, whether from one intense surgery or multiple surgeries, can cause muscle strain and result in various injuries, which may be prevented through the use of better ergonomic design and potential muscle cooling therapies.

This research suggests that muscle-cooling therapies may benefit novice and experienced surgeons in reduction of tremor and potentially increase time to functional fatigue. Due to the high variability of rates commonly reported with fatigue, the small sample size in this study makes it difficult to broadly generalize the results; however, promising results reported in this study suggest that future investigation of muscle cooling in surgical tasks would elucidate the specific effects of targeted muscle cooling. Additionally, investigation of combining limb cooling with β-blockers might further reduce tremor. Certain surgical specialties (orthopedics, vascular surgery, ophthalmology) or those surgeons who fatigue quickly (either due to overwork, age, disability, and so on) may especially benefit from further research in this area. Although many surgical journal editorials have specifically requested more longitudinal and cross-sectionals examination of aging surgeons and surgeons nearing retirement, the present trend of low sample sizes will continue to limit the capacity these studies have to influence future research.

The authors recognize the limitations within this study, the most prominent of which is generalizability beyond the sample size. Difficulty in subject recruitment resulted in only one university hospital consenting to participate, and within the hospital a large portion of the available subject pool declined to participate most notably due to time constraints. It is recognized that a surgeon's time is limited and valuable. Dedicating 2 hours for research participation with only minor compensation was not plausible for some. Future studies attempting to recruit surgeon subjects should emphasize the potential contribution to available research in addition to offering a monetary incentive. Differences in workload between experienced and novice surgeons were significant (with novice surgeons working almost 20 hours more per week, on average). However, these work hours reflect the typical workload difference between the experienced and novice surgeon populations, and one would be hard-pressed to find a surgical resident who worked less than 60 h/wk.

## References

[1] Duffield R Cooling interventions for the protection and recovery of exercise performance from exercise-induced heat stress Med Sport Sci 2008 53 89 103 19209001 10.1159/000151552

[2] Lakie M Walsh E G Arblaster L A Villagra F Roberts R C Limb temperature and human tremors J Neurol Neurosurg Psychiatry 1994 57 01 35 42 10.1136/jnnp.57.1.35 PMC485037 8301303

[3] Schenarts P J Cemaj S The aging surgeon: implications for the workforce, the surgeon, and the patient Surg Clin North Am 2016 96 01 129 138 26612025 10.1016/j.suc.2015.09.009

[4] Katlic M R Coleman J The aging surgeon Ann Surg 2014 260 02 199 201 24670863 10.1097/SLA.0000000000000667

[5] Morrison S Kavanagh J Obst S J Irwin J Haseler L J The effects of unilateral muscle fatigue on bilateral physiological tremor Exp Brain Res 2005 167 04 609 621 16078030 10.1007/s00221-005-0050-x

[6] Aoyagi Y Shephard R J Aging and muscle function Sports Med 1992 14 06 376 396 1470791 10.2165/00007256-199214060-00005

[7] Zampieri S Pietrangelo L Loefler S Lifelong physical exercise delays age-associated skeletal muscle decline J Gerontol A Biol Sci Med Sci 2015 70 02 163 173 24550352 10.1093/gerona/glu006

[8] Marino F E Methods, advantages, and limitations of body cooling for exercise performance Br J Sports Med 2002 36 02 89 94 11916888 10.1136/bjsm.36.2.89 PMC1724476

[9] Patapoutian A TRP channels and thermosensation Chem Senses 2005 30 01 i193 i194 15738110 10.1093/chemse/bjh180

[10] Cuschieri A Whither minimal access surgery: tribulations and expectations Am J Surg 1995 169 01 9 19 7818004 10.1016/s0002-9610(99)80104-4

[11] Reyes D AG Tang B Cuschieri A Minimal access surgery (MAS)-related surgeon morbidity syndromes Surg Endosc 2006 20 01 1 13 16333542 10.1007/s00464-005-0315-2

[12] Maclaren D P Gibson H Parry-Billings M Edwards R H A review of metabolic and physiological factors in fatigue Exerc Sport Sci Rev 1989 17 01 29 66 2676550

[13] Luttmann A Sökeland J Laurig W Electromyographical study on surgeons in urology. I. Influence of the operating technique on muscular strain Ergonomics 1996 39 02 285 297 8851533 10.1080/00140139608964459

[14] Slack P S Coulson C J Ma X Webster K Proops D W The effect of operating time on surgeons' muscular fatigue Ann R Coll Surg Engl 2008 90 08 651 657 18990280 10.1308/003588408X321710 PMC2727807

[15] Harwell R C Ferguson R L Physiologic tremor and microsurgery Microsurgery 1983 4 03 187 192 6669016 10.1002/micr.1920040310

[16] Humayun M U Rader R S Pieramici D J Awh C C de Juan E Jr Quantitative measurement of the effects of caffeine and propranolol on surgeon hand tremor Arch Ophthalmol 1997 115 03 371 374 9076210 10.1001/archopht.1997.01100150373010

[17] Elman M J Sugar J Fiscella R The effect of propranolol versus placebo on resident surgical performance Trans Am Ophthalmol Soc 1998 96 283 291, discussion 291–294 10360293 PMC1298399

[18] Hsu P A Cooley B C Effect of exercise on microsurgical hand tremor Microsurgery 2003 23 04 323 327 12942522 10.1002/micr.10156

